# Breeding Crops for Enhanced Food Safety

**DOI:** 10.3389/fpls.2020.00428

**Published:** 2020-04-15

**Authors:** Maeli Melotto, Maria T. Brandl, Cristián Jacob, Michele T. Jay-Russell, Shirley A. Micallef, Marilyn L. Warburton, Allen Van Deynze

**Affiliations:** ^1^Department of Plant Sciences, University of California, Davis, Davis, CA, United States; ^2^United States Department of Agriculture-Agricultural Research Service, Produce Safety and Microbiology Research, Albany, CA, United States; ^3^Western Center for Food Safety, University of California, Davis, Davis, CA, United States; ^4^Department of Plant Science and Landscape Architecture, Center for Food Safety and Security Systems, University of Maryland, College Park, MD, United States; ^5^United States Department of Agriculture-Agricultural Research Service, Corn Host Plant Research Resistance Unit Mississippi State, Starkville, MS, United States; ^6^Plant Breeding Center, Department of Plant Sciences, University of California, Davis, Davis, CA, United States

**Keywords:** food safety, crop improvement, plant breeding, enterobacterium, mycotoxins, heavy metals, human pathogens on plants, allergens

## Abstract

An increasing global population demands a continuous supply of nutritious and safe food. Edible products can be contaminated with biological (*e.g.*, bacteria, virus, protozoa), chemical (*e.g.*, heavy metals, mycotoxins), and physical hazards during production, storage, transport, processing, and/or meal preparation. The substantial impact of foodborne disease outbreaks on public health and the economy has led to multidisciplinary research aimed to understand the biology underlying the different contamination processes and how to mitigate food hazards. Here we review the knowledge, opportunities, and challenges of plant breeding as a tool to enhance the food safety of plant-based food products. First, we discuss the significant effect of plant genotypic and phenotypic variation in the contamination of plants by heavy metals, mycotoxin-producing fungi, and human pathogenic bacteria. In addition, we discuss the various factors (*i.e.*, temperature, relative humidity, soil, microbiota, cultural practices, and plant developmental stage) that can influence the interaction between plant genetic diversity and contaminant. This exposes the necessity of a multidisciplinary approach to understand plant genotype × environment × microbe × management interactions. Moreover, we show that the numerous possibilities of crop/hazard combinations make the definition and identification of high-risk pairs, such as *Salmonella*-tomato and *Escherichia coli*-lettuce, imperative for breeding programs geared toward improving microbial safety of produce. Finally, we discuss research on developing effective assays and approaches for selecting desirable breeding germplasm. Overall, it is recognized that although breeding programs for some human pathogen/toxin systems are ongoing (*e.g., Fusarium* in wheat), it would be premature to start breeding when targets and testing systems are not well defined. Nevertheless, current research is paving the way toward this goal and this review highlights advances in the field and critical points for the success of this initiative that were discussed during the Breeding Crops for Enhanced Food Safety workshop held 5–6 June 2019 at University of California, Davis.

## Introduction

The demand for nutritious and safe food will increase as the human population is expected to reach between 9.4 and 10.1 billion in 2050 and between 9.4 and 12.7 billion in 2100 (United Nations [UN], Department of Economic, and Social Affairs, Population Division., 2019a), along with increasing urbanization and standards of living (United Nations [UN], Department of Economic, and Social Affairs, Population Division, 2019b). Healthy consumption of grains, oilseeds, nuts, and fresh fruits and vegetables is part of an integrated strategy to decrease the risk for diet-related chronic diseases, such as cardiovascular disease, type 2 diabetes, some types of cancer, and obesity (U.S. Department of Health and Human Services-U.S. Department of Agriculture [HHS-USDA], 2015). However, the World Health Organization (WHO) report shows that at global level, 31 hazards caused 600 million foodborne illnesses and 420,000 deaths in 2010 (World Health Organization [WHO], 2015). Health concerns exist due to the consumption of mycotoxins produced by fungi that frequently infect grain, oilseed, and nut crops (Binder et al., 2007; Marin et al., 2013). The health burdens placed on consumers and economic burdens placed on farmers and processors by the presence of these toxins can be severe (Schaafsma, 2002; Wu, 2007; Zain, 2011). Furthermore, heavy metals (*e.g.*, cadmium and arsenic), allergens (*e.g*., actinidin and Ara h proteins), and accumulations of natural molecules and compounds (*e.g*., nitrates, cyanoglycosides, vicine and convicine, gluten, and Kunitz trypsin inhibitor) may be detrimental to human health.

The fresh market has resulted in a wide variety of fresh fruits and vegetables available throughout the year (Huang, 2013; Siegel et al., 2014). At the same time, the number of foodborne disease outbreaks related to consumption of contaminated fresh or minimally processed produce has been increasing (Nguyen et al., 2015; Bennett et al., 2018; Turner et al., 2019). In the United States, 48 million illnesses and 3000 deaths associated with food-borne diseases occur annually, with approximately one half associated with crops (Painter et al., 2013). In the European Union, during the period 2004–2012, there were 198 outbreaks linked to the consumption of fresh produce (Callejón et al., 2015). Beyond the burden on public health, foodborne illness outbreaks negatively affect the economics of the industry. It is estimated that the overall cost of food safety incidences for the economy of the United States is $7 billion per year, which comes from notifying consumers, removing food from shelves, and paying damages from lawsuits (Hussain and Dawson, 2013). Furthermore, a single produce-borne disease outbreak can trigger a sharp decrease in the market of the affected crop for years (Calvin et al., 2004; Arnade et al., 2009; Ribera et al., 2012).

Following a number of large multistate foodborne disease outbreaks linked to contaminated fresh produce^1^, the American Phytopathological Society-Public Policy Board (APS-PPB) convened the first formal activity in 2007 in a symposium titled “Cross Domain Bacteria: Emerging Threats to Plants, Humans, and Our Food Supply”^2^. A working group on “Human Pathogens on Plants” was assembled to create solutions for this problem and has since convened as a satellite meeting during annual APS meetings. Similar activities have been conducted in Europe through the COST Action on “Control of Human Pathogenic Micro-organisms in Plant Production Systems”^3^.

Leafy greens are annually involved in food safety incidents in the United States. From 1996 to 2016, 134 confirmed incidents, including 46 outbreaks, were identified to be linked to products from California (Turner et al., 2019) that provides one-third of the vegetables and two-thirds of the fruit and nuts in the United States according to the California Department of Agriculture (CDFA), California Agricultural Production Statistics^4^. During this period, lettuce and spinach were reported as the main vehicles of food safety incidents (39 and 26%, respectively; Turner et al., 2019). After three major outbreaks in 2006, the leafy green industries in Arizona and California created the Leafy Green Marketing Agreement (LGMA) with evidence-based food safety metrics that are updated to incorporate the most current state-of-the-science^5^. Likewise, the U.S. Food and Drug Administration (FDA) subsequently implemented the Food Safety Modernization Act (FSMA) to address the significant public health burden of preventable foodborne diseases. Under FSMA, the Produce Safety Rule established, for the first time, science-based minimum standards that include on-farm regulation of fresh fruits and vegetables grown for human consumption^6^.

Food safety is a complex issue that requires a concerted effort among scientists, regulators, seed/nursery industry, processors, retailers, and other stakeholders from diverse disciplines and research fields who do not often have the opportunity to meet and discuss global, comprehensive, and objective solutions. On 5–6 June 2019, the University of California, Davis hosted the first workshop on Breeding Crops for Enhanced Food Safety^7^ to identify knowledge gaps and research priorities in this emerging field to inform the USDA-NIFA and other agencies for funding and research priorities. This workshop connected plant scientists, plant breeders, extension specialists, microbiologists, and food safety experts from industry and academia to discuss collaborative efforts and multidisciplinary approaches geared toward preventing the occurrence of hazardous microbes, mycotoxins, elements, and allergens in crop and food production systems. Together, these pivotal steps by academia, industry, and government groups have laid out the opportunities to enhance food safety with plant breeding and created avenues for unique collaborative efforts and new research directions, which formed the basis for this review.

## Breeding Research to Understand the Opportunities to Reduce Food Safety Issues Associated With Crops

The presence of mycotoxins, elements, and allergens in affected food crops has a high potential for mitigation via plant breeding (Zhou et al., 2013; Warburton and Williams, 2016; Arias et al., 2018; Gaikpa and Miedaner, 2019). These substances, produced by the fungus, the plant itself, or taken up by the plant from the environment, are generally not defense compounds, but can be severely detrimental to the health of humans and animals who consume the crop in which the substances have accumulated. Crop varieties that do not support growth of the fungi that produce mycotoxins have been created in some cases (*i.e*., aflatoxin resistant maize; Williams and Windham, 2012) and heritability is sufficiently high for genetic gain in others (*e.g.*, *Fusarium* resistant wheat; Petersen et al., 2016). Additionally, it may be possible to create host plants that do not allow or create the need for the fungi to produce mycotoxins. The level of allergens in crop plants can also be reduced in some cases via plant breeding, or in others, via genetic engineering or gene editing (*e.g.*, removal of peanut allergens via transformation; Sáiz et al., 2013) and breeding for wheat varieties that do not accumulate heavy metals (Liu et al., 2019). Furthermore, breeding efforts are conducted for the reduction of antinutritional compounds, such as vicine and convicine in faba bean (Hazaei et al., 2019) and the acrylamide-forming potential of potatoes (Bethke, 2018).

Mounting evidence suggests that zoonotic bacterial pathogens of humans (*e.g*., non-typhoidal *Salmonella enterica* and *Escherichia coli* O157:H7) may have adapted to both animal and plant hosts, enabling them to survive in the food production chain (Brandl, 2006; Barak and Schroeder, 2012; Sapers and Doyle, 2014; Wiedemann et al., 2015). For example, romaine lettuce and other leafy greens continue to be linked to *E. coli* O157:H7 outbreaks traced to major leafy green production regions in Arizona and California despite widespread implementation of LGMA food safety practices; moreover, traceback and environmental assessments suggest that contamination is occurring at the pre-harvest level, but root causes remain elusive (California Department of Public Health, Emergency Response Unit [CDPH], 2010, 2014; Centers for Disease Control and Prevention [CDC], 2018; U.S. Food and Drug Administration [FDA], 2018, 2020). A few research groups have discovered phenotypic variability in the interaction between these pathogens and fresh produce, suggesting that plant genetic traits may affect plant susceptibility or tolerance to human pathogen colonization (Table 1). A complete description of the methods used in each study is listed in Supplementary Table S1. Similarly to the examples of breeding strategies described above, these reports support the basis for breeding (*i.e*., genetic variability) for decreased microbial hazards in several systems.

**TABLE 1:** A comprehensive list of studies focused on the effect of plant genotypic variation in the interaction between plants and human pathogenic bacteria.

**Classification**	**Plant genotypes**	**Pathogen genotype(s)**	**References**
Seeds	Alfalfa, fenugreek, lettuce (cultivar Iceberg), spinach, and tomato (cultivar Roma)	*Escherichia coli* serotypes O157:H7 (strains F4546, K4499, and H1730) and O104:H4 (strain BAA-2326). *Salmonella enterica* serovars Baildon, Cubana, Montevideo, and Stanley	Cui et al., 2017
Sprouts and seedlings	Alfalfa, fenugreek, lettuce (cultivar Iceberg), spinach, and tomato (cultivar Roma)	*E. coli* serotypes O157:H7 (strains F4546, K4499, and H1730) and O104:H4 (strain BAA-2326). *S. enterica* serovars Baildon, Cubana, Montevideo, and Stanley	Cui et al., 2018
	Broccoli, carrot, cilantro, endive, lettuce (cultivars Balady Aswan, Salinas 88, Little Gem, PI251246, Pavane, Valmaine, Iceberg, La Brillante, Paris Island, and Parade, Calmar), tomato (cultivars Brandywine, Amish Paste, Money Maker, Rose, Soldacki, Stupice, Green Grape, San Marzano, Nyarous, and Yellow Pear), parsley, radicchio, radish, spinach, and turnip	*S. enterica* serovars Baildon, Cubana, Eteritidis, Havana, Mbandaka, Newport, Poona, and Schwarzengrund; eight strains cocktail	Barak et al., 2008
	Lettuce (cultivars Vaila-Winter Gem, Lobjoits Green, Marshall, Little Gem, Dazzle, Unrivaled, Rosseta, Lakeland, Regina dei Ghiacci, Webbs Wonderful, Set, and Lollo Rossa)	*E. coli* serotype O157:H7 (bioluminescent strain Tn5 luxCDABE)	Quilliam et al., 2012
	Lettuce (cultivars Tamburo, Nelly, and Cancan)	*S. enterica* serovars Dublin, Typhimurium, Enteritidis, Newport, and Montevideo	Klerks et al., 2007
	Tomato (cultivars, CA Red Cherry, Heinz-1706, Moneymaker, Nyagous, Micro-Tom, Florida 91VFF, Rutgers Select, Rutgers VFA, Virginia Sweets, Plum Dandy VF. Genotypes LA4013, Movione, and Mobox)	*S. enterica* serovars Newport and Typhimurium	Han and Micallef, 2014
	Tomato (cultivars H7996, Yellow Pear, and Nyagous) and *Solanum pimpinellifolium* (cultivar WVa700)	*S. enterica* serovars Baildon, Cubana, Eteritidis, Havana, Mbandaka, Newport, Poona, and Schwarzengrund; eight strains cocktail	Barak et al., 2011
Mature leaves	Arugula, basil, lettuce (iceberg and romaine types and cultivar Ruby Red), parsley, tomato (cultivar MP1)	*S. enterica* serovar Typhimurium (strain SL1344 expressing green fluorescent protein)	Golberg et al., 2011
	Basil, cilantro, lettuce (butterhead and romaine types), and spinach	*E. coli* serotype O157:H7 (strain 86-24). *S. enterica* serovar Typhimurium (strain SL1344)	Roy and Melotto, 2019
	Cabbage (red type), lettuce (green leafy), and spinach	*S. enterica* serovars Enteritidis (strain ME18), Newport (strain 11590), and Typhimurium (strain χ3985 Δcrp-11)	Erickson and Liao, 2019
	Corn salad (cultivar Verte à coeur plein 2) and lettuce (cultivar Tizian) and	*S. enterica* serovar Typhimurium (strain 14028s)	Jechalke et al., 2019
	Lettuce (cultivars Saladin and Iceberg) and *Lactuca serriola* (accession US96UC23)	*S. enterica* serovar Senftenberg (strain 070885)	Hunter et al., 2015
	Lettuce (romaine types line RH08-0464 and cultivar Triple Threat)	*E. coli* serotype O157:H7 (strain ATCC43888)	Simko et al., 2015
	Lettuce (cultivars Gabriella, Green Star, Muir, New Red Fire, Coastal Star, Starfighter, Tropicana, and Two Star)	*E. coli* serotype O157:H7 (USDA 5, MD56, and MD58). *S. enterica* serovars Enteritidis (strain ME 18), Newport (strain 11590K), and Typhimurium (strains χ3985 Δcrp-11 and Δcya-12)	Erickson et al., 2019
	Lettuce (cultivars Salinas, Emperor, La Brillante, Lollo Rossa, Red Tide, Grand Rapids, Green Towers, and Bibb and accession 13G640-1) and *L. serriola* (accessions 12G239-1 and UC23US96)	*E. coli* serotype O157:H7 (strain 86-24). *S. enterica* serovar Typhimurium (strain 14028s)	Jacob and Melotto, 2020
	Spinach (cultivars Tyee, Space, and Bordeaux)	*E. coli* serotype O157:H7 (strains ATCC 43888, EO122, K3995, K4492, and F4546); five strains cocktail	Mitra et al., 2009
	Spinach (cultivars Emilia, Waitiki, Lazio, and Space)	*E. coli* serotype O157:H7 (strain EDL933)	Macarisin et al., 2013
	Spinach (cultivars Whale, Shasta, Barbosa, and Avenger)	*E. coli* (generic strains TVS 353, 354, and 355); individually and three strains cocktail. *E. coli* serotype O157:H7 (strains ATCC 700728 and ATCC 43888); two strains cocktail	Gutiérrez-Rodríguez et al., 2011
	Tomato (cultivars Florida Lanai, Crown Jewel, and Alisa Craig)	*S. enterica* serovar Typhimurium (strain MAE110)	Gu et al., 2013
Fruits	Cucumber (cultivars Marketmore 97, Patio Snacker, Corinto, Bella, Pepinex, and Summer Dance)	*S. enterica* serovars Newport (strain MDD 314) and Javiana (strain ATCC BAA-1593)	Callahan and Micallef, 2019
	Melon (cultivars Arava, Athena, Dulce Nectar, Jaune de Canaries, and Sivan)	*S. enterica* serovar Newport	Korir et al., 2019
	Melon (cultivars Burpee’s Ambrosia, Hale’s Best, Hearts of Gold, Israel Old Original, and Sweet ‘n Early)	*S. enterica* serovar Thompson. *E. coli s*erotype O157:H7	Duffy et al., 2008
	Melon (cultivars Oro Rico, Top Mark, and Summer Dew)	*S. enterica s*erovar Typhimurium (strain aPTVS150)	Lopez-Velasco et al., 2012a
	Melon and watermelon	*E. coli* serotype O157:H7 (strains 04P,30lC, 505B, and 5753-35); four strain cocktail	Del Rosario and Beuchat, 1995
	Melon (types cantaloupe and honeydew) and watermelon	*S. enterica* serovars Anatum, Chester, Havana, Poona, and Senftenberg); five strain cocktail	Golden et al., 1993
	Tomato (cultivars Bonny Best, Florida-47, and Solar Fire)	*S. enterica* serovar Typhimurium (strain 14028); individually. *S. enterica* serovars Javiana (strain ATCC BAA-1593), Montevideo (strain LJH519), Newport (strain C6.3), and Braenderup (strains 04E01347, 04E00783, and 04E01556); six strain cocktail	Devleesschauwer et al., 2017
	Tomato (cultivars Bonny Best, Florida-47, and Solar Fire)	*S. enterica* serovar Typhimurium (strain 14028); individually. *S. enterica* serovars Javiana (strain ATCC BAA-1593), Montevideo (strain LJH519), Newport (strain C6.3), and Braenderup (strains 04E01347, 04E00783, and 04E01556); six strain cocktail	Marvasi et al., 2013
	Tomato (cultivars, CA Red Cherry, Heinz-1706, Moneymaker, Nyagous, Micro-Tom, Florida 91VFF, Rutgers Select, Rutgers VFA, Virginia Sweets, Plum Dandy VF. Genotypes LA4013, Movione, and Mobox)	*S. enterica* serovar Typhimurium (strain LT2)	Han and Micallef, 2016
	Tomato (cultivars Alisa Craig, Amish Salad, Beefsteak, Bloody Butcher, Bonny Best, Brown Berry, Campari, Celebrity, Cocktails on Vine, Early Wonder, Florida74, Florino, Glacier, Hawaii 7997, HP/HP, John Baer, Kumato, Large Red Cherry, Mariana, Marmande, Money Maker, Never Ripe, Pearson, Red Calabash, Sebring, Snow White, Solar Fire, Solar Set, Success, Sun Gold, Tasti-Lee, and Tommy Toe)	*S. enterica* serovar Typhimurium (strain 14028); individually. *S. enterica* serovars Javiana (strain ATCC BAA-1593), Montevideo (strain LJH519), Newport (strain C6.3), and Braenderup (strains 04E01347, 04E00783, and 04E01556); six strain cocktail	Marvasi et al., 2014b
	Tomato (cultivars Bonny Best, Florida-47, and Solar Fire) and pepper (cultivar Aristotle)	*S. enterica* serovar Typhimurium (strain 14028); individually. *S. enterica* serovars Javiana (strain ATCC BAA-1593), Montevideo (strain LJH519), Newport (strain C6.3), and Braenderup (strains 04E01347, 04E00783, and 04E01556); six strain cocktail	Marvasi et al., 2015
	Tomato (cultivars Campari, Hawaii 7997, and Bonny Best)	*S. enterica* serovar Typhimurium (strain 14028)	Noel et al., 2010
	Tomato (cultivars Solar Fire and Sebring)	*S. enterica* serovar Typhimurium (strain 14028); individually. *S. enterica* serovars Javiana (strain ATCC BAA-1593), Montevideo (strain LJH519), Newport (strain C6.3), and Braenderup (strains 04E01347, 04E00783, and 04E01556); six strain cocktail	Marvasi et al., 2014a

## Plant Breeding to Address the Pre-Harvest Component of a Systems Approach Required to Manage Food Safety

Host plant resistance to the fungi that produce mycotoxins can be a synergistic part of a systems approach to reducing mycotoxins in crop plants (Grace et al., 2015). The method is economical for the farmer because it requires no additional equipment or supplies and is integral to the seed itself. It works well with other methods for controlling mycotoxins, including the use of biocontrol agents that farmers can buy and apply to the field, and proper handling and environmental conditions during harvest, drying, and storage, which can also help prevent the growth of the fungi. There has been a long history of breeding wheat for resistance to *Fusarium graminearum* that produces deoxynivalenol (DON; Moghimi et al., 2019), although complete resistance remains elusive. Significant progress has also been made, for example, in pre-breeding germplasm in maize that does not support the production of aflatoxin or significantly reduces it compared to conventional maize varieties. These traits are being introgressed into U.S. maize inbreds by Paul Williams and Marilyn Warburton at USDA–ARS, Mississippi, and Seth Murray and Wenwei Xu at Texas A&M University. Heritable plant traits that reduce the numbers of harmful human pathogen cells on the edible portions of the plant may also be incorporated into a system designed to reduce risk from these microorganisms without negatively influencing the other components of the system. Similarly, Charlie Brummer and Allen Van Deynze, with support from Richard Smith (University of California, Davis) have identified and are breeding lines of spinach that have reduced accumulation of cadmium, a heavy metal found in some soils in California that can have chronic health effects, especially in children. Wheat varieties that accumulate low levels of cadmium are being developed using the latest genomic and phenotyping technologies (Liu et al., 2019).

The fresh produce industry faces several major challenges related to controlling risks from in-field contamination of crops by zoonotic enteric pathogens (Beuchat, 1996; Cooley et al., 2007; Jay-Russell, 2013; Jeamsripong et al., 2019). First, zoonotic fecal-borne pathogens may be widespread in the environment, but rarely detected in field crop, thus making it difficult to precisely define the most important direct and indirect routes of contamination (e.g., agriculture water, animal intrusion, bioaerosols, soil amendments, etc.). Second, if bacterial contamination occurs in the field, there is no subsequent “kill step” for many popular produce items such as salad greens that are consumed raw or minimally processed. Third, the infectious dose for these pathogens may be low, especially among vulnerable populations such as young children (Tuttle et al., 1999). Although it may seem improbable that a low level of in-field contamination could result in large numbers of human foodborne illnesses, Danyluk and Schaffner (2011) developed a quantitative risk assessment model that predicted that exposure to levels of *E. coli* O157:H7 in the field—as low as -1 log CFU/g and 0.1% prevalence—could result in a nationwide outbreak in combination with postharvest contributing factors such as cross-contamination during the washing process.

These challenges underscore the critical need to identify novel approaches to prevent or reduce the public health risk from pre-harvest microbial contamination of fresh produce. Although to date, no breeding program has adopted strategies to control human pathogens on fresh produce, a few studies have taken steps in this direction. For instance, Shirley Micallef (University of Maryland) is exploring cultivar variability in fatty acid content in tomato fruit as a means to reduce the favorability of tomato fruit for *Salmonella* (Han and Micallef, 2016). Maeli Melotto (University of California, Davis) is screening lettuce germplasm for susceptibility or tolerance to *E. coli* O157:H7 and *S. enterica* to define the genetic basis for the persistence of these pathogens in leafy vegetables (Jacob and Melotto, 2020). Additionally, in collaborative studies with USDA-ARS, Salinas, CA, United States and FDA-CFSAN, Laurel, MD, Maria Brandl (USDA-ARS, Albany, CA, United States) has been investigating lettuce cultivars in relation to basal plant defense responses to plant pathogen infection and to processing for their role in enteric pathogen colonization (Simko et al., 2015; Leonard et al., unpublished).

## Research to Define and Focus on High-Risk Crop–Hazard Pairs

Given the complexity of produce safety issues and the need to prioritize efforts for the highest impact, a logical step would be to identify the crop–hazard pairs (*e.g*., human pathogen–fresh produce) that create the largest burden on public health and the economy. Typically, the severity of an outbreak is estimated by the number of illnesses, hospitalizations, and deaths. With a hazard × occurrence (probability of infection or accumulation of toxin) risk model, one can begin classifying crop/hazard pairs. Although these are relevant metrics, it is very difficult to calculate the relative risk of each crop–hazard pair due to the low re-occurrence of particular pairs associated with outbreak events and the need to accumulate a substantial amount of data over extended periods of time (sometimes decades). Nonetheless, potential targets for plant breeding that are being identified may be the basis of future research to reduce human pathogens, mycotoxins, heavy metals, toxic elements, and allergens in foods.

**FIGURE 1:**
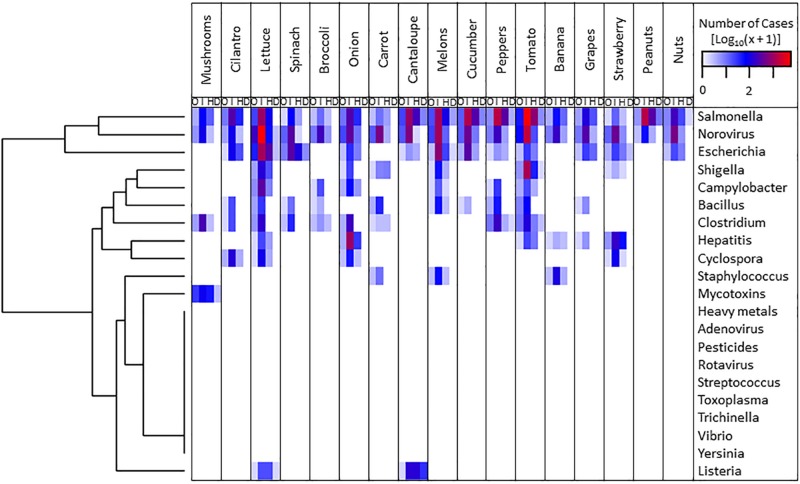
Number of outbreak (O), illness (I), hospitalization (H), and death (D) episodes of human diseases caused by the consumption of fresh produce contaminated with different etiological agents between 1998 and 2017 in the United States, according to the National Outbreak Reporting System database (https://www.cdc.gov/nors/index.html). Data were transformed with the Log_10_(*x* + 1) function. The plot was constructed with the heatmap.2 package of R using hierarchical clustering analysis for etiological agents.

Currently, the National Outbreak Reporting System (NORS^8^) of the Centers for Disease Control and Prevention reports disease outbreaks in the United States and maintains a comprehensive searchable database with information spanning from 1998 to 2017. Using this resource, we have generated a heatmap illustrating the relative importance of the major fresh produce in combination with reported etiological agents of outbreaks (Figure 1 and accompanying raw data in Supplementary Table S2). Hierarchical clustering analysis (R heatmap.2 package) of the etiological agents revealed *Salmonella*, Norovirus, and *Escherichia* as the three most important biological hazards based on the number of outbreaks, illnesses, hospitalizations, and deaths (Figure 1). In addition, the compilation of these data has enabled the identification of high priority pairs (*i.e.*, lettuce–*E. coli* O157:H7, tomato–*Salmonella*, melon–*Salmonella*, and melon–*Listeria*) for breeding programs geared toward improving microbial safety of produce (Figure 1). These systems have been studied at the genetic level by Jeri Barak (University of Wisconsin-Madison), Maria Brandl (USDA, ARS, Albany, CA, United States), Maeli Melotto (University of California, Davis), and Shirley Micallef (University of Maryland, College Park). For instance, it has been discovered that certain varieties of tomato (Marvasi et al., 2013, 2014b; Han and Micallef, 2014), lettuce (Klerks et al., 2007; Quilliam et al., 2012; Simko et al., 2015; Jacob and Melotto, 2020), cucumbers (Callahan and Micallef, 2019), and melons (Korir et al., 2019) are less likely to support pathogen populations than others, suggesting a plant genetic component underlying these traits (Table 1 and Supplementary Table S1). Bacterial serotype and strain specificities to plants have also been uncovered (Klerks et al., 2007; Cui et al., 2018; Erickson and Liao, 2019; Wong et al., 2019). Identifying the molecular mechanisms underlying these interactions can point to promising plant traits to further explore and integrate in plant breeding programs. Encouraging commercial production of plant varieties that carry relevant traits without compromising other aspects of plant productivity and product marketing might help reduce illness from produce.

In the area of mycotoxin contamination, *Fusarium* in wheat is an annual occurrence with prevalence determined by local weather at crop maturity (GIPSA, 2006). Aflatoxin in maize is regional and limited to more hot and humid regions, but remains relatively low in the main U.S. corn belt. However, on a global scale, up to 80% of maize seed lots can be contaminated in tropical areas such as Sub-Saharan Africa and India (GIPSA, 2006). Peanuts have similar occurrence of aflatoxin in areas such as East Africa. Heavy metals are predicted to continue to be a problem as arable land becomes increasingly scarce due to desertification and urbanization, and lands or irrigation water with heavy metals are more extensively used (Arora et al., 2008). These hazards can also be prioritized and paired with the crops in which the highest occurrence makes them the greatest human health hazards (Figure 1).

## Multidisciplinary Approach to Understanding Plant Genotype × Environment × Microbe × Management Interactions

A multidisciplinary approach will be necessary to develop plant breeding research programs since the occurrence of a contamination event depends on the interaction of several factors such as plant genotype, environmental conditions, the microbe and its community, and plant management practices. Together, these variables may create “The Perfect Storm.” Interactions between enteric pathogens and plants affect all mitigation strategies aimed at inhibiting pathogen growth and survival on crops to improve their microbial safety. Below, we discuss various hurdles and important aspects of these interactions that must be considered to ensure the success of a plant breeding program for enhanced crop safety.

One of the most significant challenges in breeding crops to decrease the risk of contamination with enteric pathogens is that they have lower fitness on plants than most well-characterized plant commensal and pathogenic bacterial species. Nevertheless, given the recurrence of food-borne illness outbreaks linked to produce (Figure 1), the ability of enteric pathogens to multiply and survive as epiphytes and endophytes implies that particular plant phenotypes and genotypes can affect their fitness in the plant habitat (Table 1). For example, the composition of substrates available on fruit and leaf surfaces as well as in their internal tissue (Brandl and Amundson, 2008; Marvasi et al., 2014b; Crozier et al., 2016; Han and Micallef, 2016); the density of trichomes, stomata, and veins (Barak et al., 2011; Kroupitski et al., 2011; Macarisin et al., 2013; Jacob and Melotto, 2020), which harbor larger pools of substrates than other areas of leaves; and the physical and chemical composition of the cuticle layer on various parts of the plant (Lima et al., 2013; Hunter et al., 2015), which affects water dispersal and hence, water and nutrient availability to microbial inhabitants (Marcell and Beattie, 2002), may all be relevant traits to investigate in plant breeding efforts for their effect on enteric pathogen colonization.

Temperature and humidity conditions, and the presence of free water, are important in the multiplication and survival of enteric pathogens (Brandl and Mandrell, 2002; Brandl et al., 2004; Stine et al., 2005; Fonseca et al., 2011; Deblais et al., 2019; Roy and Melotto, 2019) and must be investigated simultaneously with the role of other plant traits. This includes consideration of agricultural practices, such as irrigation type and frequency (Fonseca et al., 2011; Williams et al., 2013; Castro-Ibáñez et al., 2015; Moyne et al., 2019), which may greatly affect the success of any breeding strategy aimed at reducing surface and internal plant colonization by food-borne pathogens. It is also clear that physicochemical stressors in the plant environment (*e.g*., desiccation and UV radiation) (Jacobs and Sundin, 2001; Lindow and Brandl, 2003; Poza-Carrion et al., 2013) may overshadow other factors in their inhibitory effect on enteric pathogens. Therefore, the role of certain heritable plant traits at microsites that shield the bacterial cells from such fatal stressors should be investigated at the microscopic level as well as the plant or tissue level.

Fully elucidating the interaction between food safety-relevant microbes and crops necessitates the consideration of the entire plant microbiome below and above ground. Plant microbiota are complex and strongly driven by plant genetics, plant age, plant anatomical structure, and environmental factors (Lopez-Velasco et al., 2011; Rastogi et al., 2012; Ottesen et al., 2013; Lima et al., 2013; Williams et al., 2013; Yu et al., 2018). Identifying conditions that select for members of the plant microbiota able to competitively exclude enteric pathogens, which in general exhibit reduced fitness in the plant niche, can form an important component of this phytobiome approach (Cooley et al., 2003, 2006; Lopez-Velasco et al., 2012b; Williams et al., 2013; López-Gálvez et al., 2018). In addition, rhizosphere and phyllosphere microbial communities can comprise epiphytes (including pathogenic species) known to affect plant colonization by enteric pathogens or toxigenic fungi either antagonistically through biocontrol strategies or favorably by supporting survival and growth. For instance, phytopathogens that actively degrade plant tissue or trigger plant chlorosis and necrosis may cause changes in pH and nutrient levels that favor the establishment and proliferation of enteric pathogens (Brandl, 2008; Goudeau et al., 2013; Potnis et al., 2014, 2015; Simko et al., 2015; George et al., 2018). Adjustment of management practices and environmental conditions to modulate and exploit microbe–microbe interactions should be actively investigated as part of a holistic approach to inhibit or prevent the colonization of enteric pathogens on/in plants.

Certain plant phenotypes may have independent as well as co-dependent effects with other plant features so that their role may only be fully revealed by actively investigating and/or selecting for both traits simultaneously. For example, entry of enteric pathogens into the plant tissue, where they are shielded from external environmental stressors, is thought to increase their survival in the plant habitat (Kroupitski et al., 2009; Erickson, 2012; Roy et al., 2013; Roy and Melotto, 2019). Thus, selecting for genotypes with lower stomatal density and stomatal pore size (Jacob and Melotto, 2020) may prove to be effective in reducing the probability of pathogen survival on plants in the field, provided that plant productivity is not impacted by the selection of that trait. Furthermore, basal plant defense responses to the presence of human pathogens (Thilmony et al., 2006; Schikora et al., 2011; Garcia et al., 2014), which can only take place upon exposure of plant cells to, and close interaction with, microbial cells in the plant apoplast, require entry of the enteric pathogen cells into the substomatal space of the tissue. Consequently, the full potential of breeding for a cultivar that is less hospitable to the endophytic lifestyle of an enteric pathogen may require consideration of both plant traits, *i.e*., traits that affect the entry of the pathogen cells into the plant (Oblessuc et al., 2019) and those that affect the plant response once the cells have gained entry (Jacob and Melotto, 2020; Oblessuc et al., 2020).

The role of the physiological state of plants in their interaction with enteric pathogens cannot be understated. Plant defense responses may vary depending on the age of the plant tissue, the overall plant age, challenge history, and association with other microbes such as plant growth promoting rhizobacteria and plant pathogens (Simko et al., 2015; Bernstein et al., 2017; Hsu and Micallef, 2017). The carrying capacity of plant tissue for enteric pathogens depends on plant species and cultivar, leaf age, fruit ripeness, and root age given that structure and opening density via cracks at the secondary root emergence sites change over time (Table 1). Evidence is increasing that changes in temperature and rainfall caused by climate change may affect plant physiological and anatomical responses. These include stomatal conductance and density, leaf area and cuticle thickness, plant morphology, and plant nutrient cycling (Wang et al., 2008; Fraser et al., 2009; Cornelissen and Makoto, 2014). The level of relative humidity can significantly influence stomatal movement that can affect colonization of the leaf interior by human pathogenic bacteria (Roy and Melotto, 2019). It is clear that if these are targets of breeding programs for improving food safety, these traits will have to be resilient under long-term shift in weather patterns. Enteric pathogens vary broadly in their fitness as epiphytes and endophytes in a species-specific manner, and even based on variation at the inter- and intra-strain level (Klerks et al., 2007; Cui et al., 2018; Erickson and Liao, 2019; Wong et al., 2019). In particular, surface appendages, such as different types of fimbriae and adhesins that act as important plant attachment factors or flagella and other surface molecules that may trigger defense signaling cascades, vary among and within enteric species and strains (Lapidot and Yaron, 2009; Macarisin et al., 2012; Seo and Matthews, 2012; Roy et al., 2013; Garcia et al., 2014; Carter et al., 2018). Preferential bacterial pathogenic species and even serotype-commodity pairs are not uncommon and the basis for this specificity is still poorly understood. Clearly, phenotypic and genotypic variation among food-borne pathogen targets must also be taken into account while selecting for plant targets to enhance microbial crop safety.

Domestication of several crops has resulted in desirable agronomic and organoleptic traits such as shape, color, and prolonged shelf-life, with the unintended loss of other traits (Gepts, 2014; Purugganan, 2019). The resulting loss in genetic variation may have reduced the ability of some crops to cope with fluctuating environmental conditions and biotic challenges (Chen et al., 2015; Brisson et al., 2019). Despite this, genetic diversity could still reside in germplasm that is not commercially grown (such as traditional varieties, landraces, and crop wild relatives), allowing for the possibility of reintroducing genotypic and phenotypic traits that restore lost properties or establish new ones (Tanksley and McCouch, 1997; Smale and Day-Rubenstein, 2002). The underlying genetic basis for traits that enhance food safety are largely unknown, but as more research uncovers the interactions between plant, pathogen, and the environment, opportunities for identifying these traits will increase.

Traits that confer enhanced food safety are likely complex and controlled by multiple genes, presenting challenges to breeding efforts, especially for human pathogen–plant interactions. A starting point could be genome-wide association studies followed by metabolic pathway analysis (Li et al., 2019; Thrash et al., 2020) or functional analysis of mapped intervals (Korte and Farlow, 2013; Bartoli and Roux, 2017). For instance, one could predict various biochemical pathways needed for the synthesis of secondary metabolites with antioxidant and antimicrobial properties that could influence plant-microbe interactions and plant responses to associated microbiota. These interactions may be extremely important in food safety and should be a major focus of pre-breeding efforts.

Given the overall challenge of considering numerous aspects of plant genotype × environment × microbe × management interactions, a concerted effort to focus on given pathogen–crop models may be necessary to make headway in utilizing plant breeding as a feasible strategy to enhance produce safety. For effective genetic gain, a systems approach that maximizes consistency and differentiation of the desired phenotypes is essential. These traits must be considered with major traits of crop yield, quality, and resistance to abiotic and biotic stresses.

## Research on Developing Effective Assays and Approaches for Selecting Desirable Breeding Germplasm

Microbial food safety issues are rare events and tracking the source of disease outbreaks is extremely complex, making it difficult to predict or determine their cause (Slayton et al., 2011; Taylor et al., 2013; Hu et al., 2016). Thus, the best way to minimize these events is to perform risk assessment analyses (Uyttendaele et al., 2016; Pang et al., 2017). As discussed above, it has become evident that the plant is not a passive vehicle for microbial food hazards, hence providing opportunities to breed crops for enhanced food safety. The challenge remains to identify effective traits and genetic variability useful for breeding.

It has long been possible to breed plant germplasm that is resistant to plant pathogens. For example, the *Fusarium* pathogen synthesizes toxic DON and/or fumonisins and reduces seed set and fill in wheat; *Aspergillus flavus* can cause ear rots of maize in environmental conditions suitable for fungal growth. In both cases, these fungi can reduce plant yield and germplasm resistant to these pathogens is available (Petersen et al., 2016; Pekar et al., 2019). However, in cases where the fitness of the plant is not as directly reduced by the presence of the pathogen, traits that could potentially increase food safety may be harder to find and may require indirect or more creative solutions. They also compete with priorities for crop production and quality in breeding programs.

Edible plants carrying human pathogens generally do not show visual symptoms as they would when infected with plant pathogens, particularly when they occur at low levels^9^. This creates a challenge in developing screening assays to identify phenotypes with useful variation to support breeding efforts. Unlike the challenges associated with microbial hazards, detection of elements such as nitrates or heavy metals (*e.g*., cadmium) is relatively easy with standard tissue analysis. Allergens can often be detected by routine (but still somewhat costly) assays (Senyuva et al., 2019). However, for human pathogens, rapid and cost-effective assays still need to be developed for routine screening of breeding populations, although some efforts have been made in this direction (Jacob and Melotto, 2020)^9^. These assays will allow large scale assessment of germplasm to find the best expression of useful traits and their introgression into cultivated varieties. Despite the challenges, variations in human pathogen colonization of lettuce, tomato, and spinach genotypes have already been determined.

An additional hurdle comes from the fact that microbial colonization is a complex behavior influenced by the plant host–pathogen combination and crop management practice such as irrigation type and crop fertilization (Fonseca et al., 2011; Marvasi et al., 2014a). Human pathogen–plant models should be developed for the purpose of breeding efforts to enhance food safety based on enteric pathogen strain–plant commodity variety pairs identified from prominent or recurring foodborne illness outbreaks. At the same time, plant genetic resources that may facilitate genome-wide association studies should not be excluded. Furthermore, the use of human pathogens in routine assays requires highly trained personnel and laboratory/greenhouse biosafety conditions according to NIH guidelines, in addition to considerable costs associated with the handling of microbial hazards in contained facilities. These approaches will require collaborative efforts among food safety experts, plant–microbe interaction biologists, microbiologists, and crop breeders for successful advancements in the field.

## Conclusion and Recommendations

It is generally recognized that although breeding programs for certain human pathogen/toxin systems are ongoing (*e.g., Fusarium* in wheat), it would be premature to engage in plant breeding for other aspects of food safety for which targets and testing systems are not yet well defined. Nevertheless, current research is paving the way toward this goal. To ensure advances in the field, the following points are critical to the success of this initiative:

(1) Continue foundational research to generate crucial knowledge of plant interactions with human pathogens and contamination of food with microbes, mycotoxins, elements, and allergens.
(2) Initiate pre-breeding screening strategies to characterize genetic variability, heritability, and efficacy of target traits.
(3) Support breeding programs where genetic variation and efficacy is established (*e.g.*, breeding lines that accumulate less aflatoxins or heavy metals).

Ultimately, in combination with agricultural practices and interventions, the recognition that plant breeding for enhanced food safety can be another layer in our fight to reduce foodborne illness associated with crops necessitates research goals and funding prioritization to enable advances in this area.

## Author Contributions

All authors have contributed to the writing of the review.

## Conflict of Interest

The authors declare that the research was conducted in the absence of any commercial or financial relationships that could be construed as a potential conflict of interest.

## Footnotes

1 https://www.cdc.gov/foodsafety/outbreaks/multistate-outbreaks/outbreaks-list.html

2 http://www.apsnet.org/members/outreach/ppb/Pages/FoodSafety.aspx

3 http://www.cost.eu/COST_Actions/ca/CA16110?

4 https://www.cdfa.ca.gov/statistics/

5 https://lgma.ca.gov/food-safety-program

6 https://www.fda.gov/food/food-safety-modernization-act-fsma/fsma-final-rule-produce-safety

7 https://melotto.ucdavis.edu/nifa-workshop-2019

8 https://www.cdc.gov/nors/

9 Oblessuc, P. R., and Melotto, M. (under review). A simple assay to assess *Salmonella enterica* persistence in lettuce leaves after a low inoculation dose. *Front. Plant Sci.*

## References

[B1] Arias R. S. Sobolev V. S. Massa A. N. Orner V. A. Walk T. E. Ballard L. L. (2018). New tools to screen wild peanut species for aflatoxin accumulation and genetic fingerprinting. *BMC Plant Biol.* 18:170. 10.1186/s12870-018-1355-9 30111278 PMC6094572

[B2] Arnade C. Calvin L. Kuchler F. (2009). Consumer response to a food safety shock: the 2006 food-borne illness outbreak of *E. coli* O157:H7 linked to spinach. *Rev. Agric. Econ.* 31 734–750. 10.1111/j.1467-9353.2009.01464.x

[B3] Arora M. Kiran B. Rani S. Rani A. Kaur B. Mittal N. (2008). Heavy metal accumulation in vegetables irrigated with water from different sources. *Food Chem.* 111 811–815. 10.1016/j.foodchem.2008.04.049 29100695

[B4] Barak J. D. Kramer L. C. Hao L. (2011). Colonization of tomato plants by *Salmonella enterica* is cultivar dependent, and type 1 trichomes are preferred colonization sites. *Appl. Environ. Microbiol.* 77 498–504. 10.1128/AEM.01661-10 21075871 PMC3020540

[B5] Barak J. D. Liang A. Narm K. E. (2008). Differential attachment to and subsequent contamination of agricultural crops by *Salmonella enterica*. *Appl. Environ. Microbiol.* 74 5568–5570. 10.1128/AEM.01077-08 18606796 PMC2546622

[B6] Barak J. D. Schroeder B. K. (2012). Interrelationships of food safety and plant pathology: the life cycle of human pathogens on plants. *Annu. Rev. Phytopathol.* 50 241–266. 10.1146/annurev-phyto-081211-172936 22656644

[B7] Bartoli C. Roux F. (2017). Genome-wide association studies in plant pathosystems: toward an ecological genomics approach. *Front. Plant Sci.* 8:763. 10.3389/fpls.2017.00763 28588588 PMC5441063

[B8] Bennett S. D. Sodha S. V. Ayers T. L. Lynch M. F. Gould L. H. Tauxe R. V. (2018). Produce-associated foodborne disease outbreaks, USA, 1998–2013. *Epidemiol. Infect.* 11 1397–1406. 10.1017/S0950268818001620 PMC9133681 29923474

[B9] Bernstein N. Sela Saldinger S. Dudai N. Gorbatsevich E. (2017). Salinity stress does not affect root uptake, dissemination and persistence of *Salmonella* in sweet-basil (*Ocimum basilicum*). *Front. Plant Sci.* 8:675 10.3389/fpls.2017.00675 PMC5411819 28512466

[B10] Bethke P. C. (2018). Progress and successes of the specialty crop research initiative on acrylamide reduction in processed potato products. *Am. J. Potato Res.* 95 328–337. 10.1007/s12230-018-9660-2

[B11] Beuchat L. R. (1996). Pathogenic microorganisms associated with fresh produce. *J. Food Prot.* 59 204–216. 10.4315/0362-028X-59.2.204 31159004

[B12] Binder E. M. Tan L. M. Chin L. J. Handl J. Richard J. (2007). Worldwide occurrence of mycotoxins in commodities, feeds and feed ingredients. *Anim. Feed Sci. Technol.* 137 265–282. 10.1016/j.anifeedsci.2007.06.005 24786003

[B13] Brandl M. T. (2006). Fitness of human enteric pathogens on plants and implications for food safety. *Annu. Rev. Phytopathol.* 44 367–392. 10.1146/annurev.phyto.44.070505.143359 16704355

[B14] Brandl M. T. (2008). Plant lesions promote the rapid multiplication of *Escherichia coli* O157:H7 on postharvest lettuce. *Appl. Environ. Microbiol.* 74 5285–5289. 10.1128/AEM.01073-08 18641153 PMC2546645

[B15] Brandl M. T. Amundson R. (2008). Leaf age as a risk factor in contamination of lettuce with *Escherichia coli* O157:H7 and *Salmonella enterica*. *Appl. Environ. Microbiol.* 74 2298–2306. 10.1128/AEM.02459-07 18310433 PMC2293143

[B16] Brandl M. T. Haxo A. F. Bates A. H. Mandrell R. E. (2004). Comparison of survival of *Campylobacter jejuni* in the phyllosphere with that in the rhizosphere of spinach and radish plants. *Appl. Environ. Microbiol.* 70 1182–1189. 10.1128/aem.70.2.1182-1189.2004 14766604 PMC348832

[B17] Brandl M. T. Mandrell R. E. (2002). Fitness of *Salmonella enterica* serovar Thompson in the cilantro phyllosphere. *Appl. Environ. Microbiol.* 68 3614–3621. 10.1128/AEM.68.7.3614-3621.2002 12089050 PMC126799

[B18] Brisson V. L. Schmidt J. E. Northen T. R. Vogel J. P. Gaudin A. C. M. (2019). Impacts of maize domestication and breeding on rhizosphere microbial community recruitment from a nutrient depleted agricultural soil. *Sci. Rep.* 9:15611. 10.1038/s41598-019-52148-y 31666614 PMC6821752

[B19] California Department of Public Health, Emergency Response Unit [CDPH] (2010). *Investigation of the Escherichia coli O157:H7 Outbreak Associated with Iceberg Lettuce.* Sacramento, CA: California Department of Public Health, Food and Drug Branch.

[B20] California Department of Public Health, Emergency Response Unit [CDPH] (2014). *Environmental Investigation of Escherichia coli O157:H7 Outbreak in October 2013 Associated with Pre-Packaged Salads.* Sacramento, CA: California Department of Public Health, Food and Drug Branch.

[B21] Callahan M. T. Micallef S. A. (2019). Waxing and cultivar affect *Salmonella enterica* persistence on cucumber (*Cucumis sativus* L.) fruit. *Int. J. Food Microbiol.* 310:108359. 10.1016/j.ijfoodmicro.2019.108359 31655448

[B22] Callejón R. M. Rodríguez-Naranjo M. I. Ubeda C. Hornedo-Ortega R. Garcia-Parrilla M. C. Troncoso A. M. (2015). Reported foodborne outbreaks due to fresh produce in the United States and European Union: trends and causes. *Foodborne Pathog. Dis.* 12 32–38. 10.1089/fpd.2014.1821 25587926

[B23] Calvin L. Avendaño B. Schwentesius R. (2004). *The Economics of Food Safety: The Case of Green Onions and Hepatitis A Outbreaks. Economic Research Service/USDA VGS-305-01.* Available online at: https://www.ers.usda.gov/publications/vgs/nov04/VGS30501/VGS30501.pdf (accessed November 20, 2012).

[B24] Carter M. Q. Brandl M. T. Kudva I. T. Katani R. Moreau M. R. Kapur V. (2018). Conditional function of autoaggregative protein *Cah* and common *cah* mutations in Shiga toxin-producing *Escherichia coli*. *Appl. Environ. Microbiol.* 84:e01739-17. 10.1128/AEM.01739-17 29054868 PMC5734025

[B25] Castro-Ibáñez I. Gil M. I. Tudela J. A. Ivanek R. Allende A. (2015). Assessment of microbial risk factors and impact of meteorological conditions during production of baby spinach in the Southeast of Spain. *Food Microbiol.* 49 173–181. 10.1016/j.fm.2015.02.004 25846928

[B26] Centers for Disease Control and Prevention [CDC] (2018). *Multistate outbreak of E. coli O157:H7 Infections Linked To Romaine Lettuce (final Update).* Available online at: https://www.cdc.gov/ecoli/2018/o157h7-04-18/index.html (accessed January 24, 2020).

[B27] Chen Y. J. Gols R. Benrey B. (2015). Crop domestication and its impact on naturally selected trophic interactions. *Rev. Entomol.* 60 35–58. 10.1146/annurev-ento-010814-020601 25341108

[B28] Cooley M. Carychao D. Crawford-Miksza L. Jay M. T. Myers C. Rose C. (2007). Incidence and tracking of *Escherichia coli* O157:H7 in a major produce production region in California. *PLoS One* 2:e1159. 10.1371/journal.pone.0001159 18174909 PMC2174234

[B29] Cooley M. B. Chao D. Mandrell R. E. (2006). *Escherichia coli* O157:H7 survival and growth on lettuce is altered by the presence of epiphytic bacteria. *J. Food Prot.* 69 2329–2335. 10.4315/0362-028x-69.10.2329 17066909

[B30] Cooley M. B. Miller W. G. Mandrell R. E. (2003). Colonization of *Arabidopsis thaliana* with *Salmonella enterica* and enterohemorrhagic *Escherichia coli* O157:H7 and competition by *Enterobacter asburiae*. *Appl. Environ. Microbiol.* 69 4915–4926. 10.1128/AEM.69.8.4915-4926.2003 12902287 PMC169118

[B31] Cornelissen J. H. C. Makoto K. (2014). Winter climate change, plant traits and nutrient and carbon cycling in cold biomes. *Ecol. Res.* 29 517–527. 10.1007/s11284-013-1106-1

[B32] Crozier L. Hedley P. E. Morris J. Wagstaff C. Andrews S. C. Toth I. (2016). Whole-Transcriptome analysis of verocytotoxigenic *Escherichia coli* O157:H7 (Sakai) suggests plant-species-specific metabolic responses on exposure to spinach and lettuce extracts. *Front. Microbiol.* 7:1088. 10.3389/fmicb.2016.01088 27462311 PMC4940412

[B33] Cui Y. Liu D. Chen J. (2018). Fate of various *Salmonella enterica* and enterohemorrhagic *Escherichia coli* cells attached to alfalfa, fenugreek, lettuce, and tomato seeds during germination. *Food Control* 88 229–235. 10.1016/j.foodcont.2018.01.011

[B34] Cui Y. Walcott R. Chen J. (2017). Differential attachment of *Salmonella enterica* and enterohemorrhagic *Escherichia coli* to alfalfa, fenugreek, lettuce, and tomato seeds. *Appl. Environ. Microbiol.* 83:AEM.03170-16. 10.1128/AEM.03170-16 28130295 PMC5359487

[B35] Danyluk M. D. Schaffner D. W. (2011). Quantitative assessment of the microbial risk of leafy greens from farm to consumption: preliminary framework, data, and risk estimates. *J. Food Prot.* 74 700–708. 10.4315/0362-028X.JFP-10-373 21549039

[B36] Deblais L. Helmy Y. A. Testen A. Vrisman C. Jimenez Madrid A. M. Kathayat D. (2019). Specific environmental temperature and relative humidity conditions and grafting affect the persistence and dissemination of *Salmonella enterica* subsp. *enterica* serotype Typhimurium in tomato plant tissues. *Appl Environ. Microbiol.* 85:e00403-19. 10.1128/AEM.00403-19 30926732 PMC6532026

[B37] Del Rosario B. A. Beuchat L. R. (1995). Survival and growth of enterohemorrhagic *Escherichia coli* 0157:H7 in cantaloupe and watermelon. *J. Food Prot.* 58 105–107. 10.4315/0362-028X-58.1.105 31121778

[B38] Devleesschauwer B. Marvasi M. Giurcanu M. C. Hochmuth G. J. Speybroeck N. Havelaar A. H. (2017). High relative humidity pre-harvest reduces post-harvest proliferation of *Salmonella* in tomatoes. *Food Microbiol.* 66 55–63. 10.1016/j.fm.2017.04.003 28576373

[B39] Duffy B. Ravva S. Stanker L. (2008). Cantaloupe cultivar differences as opportunistic hosts for human pathogenic *Escherichia coli* O157:H7 and *Salmonella*. *Eur. J. Hort. Sci.* 73 73–75.

[B40] Erickson M. C. (2012). Internalization of fresh produce by foodborne pathogens. *Annu. Rev. Food Sci. Technol.* 3 283–310. 10.1146/annurev-food-022811-101211 22243280

[B41] Erickson M. C. Liao J. Y. (2019). Variation in recovery of *Salmonella* strains extracted from leafy greens. *LWT Food Sci. Technol.* 107 185–190. 10.1016/j.lwt.2019.02.078

[B42] Erickson M. C. Liao J. Y. Payton A. S. Cook P. W. Den Bakker H. C. Bautista J. (2019). Pre-harvest internalization and surface survival of *Salmonella* and *Escherichia coli* O157:H7 sprayed onto different lettuce cultivars under field and growth chamber conditions. *Int. J. Food Microbiol.* 16 197–204. 10.1016/j.ijfoodmicro.2018.12.001 30551016

[B43] Fonseca J. M. Fallon S. D. Sanchez C. A. Nolte K. D. (2011). *Escherichia coli* survival in lettuce fields following its introduction through different irrigation systems. *J. Appl. Microbiol.* 110 893–902. 10.1111/j.1365-2672.2011.04942.x 21214696

[B44] Fraser L. H. Greenall A. Carlyle C. Turkington R. Friedman C. R. (2009). Adaptive phenotypic plasticity of *Pseudoroegneria spicata*: response of stomatal density, leaf area and biomass to changes in water supply and increased temperature. *Ann. Bot.* 103 769–775. 10.1093/aob/mcn252 19088084 PMC2707864

[B45] Gaikpa D. S. Miedaner T. (2019). Genomics-assisted breeding for ear rot resistances and reduced mycotoxin contamination in maize: methods, advances and prospects. *Theor. Appl. Genet.* 132 2721–2739. 10.1007/s00122-019-03412-2 31440772

[B46] Garcia A. V. Charrier A. Schikora A. Bigeardm J. Pateyron S. de Tauzia-Moreau M. L. (2014). Salmonella enterica flagellin is recognized via FLS2 and activates PAMP-Triggered Immunity in *Arabidopsis thaliana*. *Mol. Plant* 7 657–674. 10.1093/mp/sst145 24198231

[B47] George A. S. Cox C. E. Desai P. Porwollik S. Chu W. de Moraes M. H. (2018). Interactions of *Salmonella enterica* serovar Typhimurium and *Pectobacterium carotovorum* within a tomato soft rot. *Appl. Environ. Microbiol.* 84:e01913-17. 10.1128/AEM.01913-17 29247060 PMC5812938

[B48] Gepts P. (2014). The contribution of genetic and genomic approaches to plant domestication studies. *Curr. Opin. Plant Biol.* 18 51–59. 10.1016/j.pbi.2014.02.001 24631844

[B49] Gipsa. (2006). *Grain Fungal Diseases & Mycotoxin Reference*, 2nd Edn Kansas City: USDA.

[B50] Golberg D. Kroupitski Y. Belausov E. Pinto R. Sela S. (2011). *Salmonella* Typhimurium internalization is variable in leafy vegetables and fresh herbs. *Int. J. Food Microbiol.* 145 250–257. 10.1016/j.ijfoodmicro.2010.12.031 21262550

[B51] Golden D. A. Rhodehamel E. F. Kautter D. A. (1993). Growth of *Salmonella* spp. in cantaloupe, watermelon, and honeydew melons. *J. Food Prot.* 56 194–196. 10.4315/0362-028X-56.3.194 31084082

[B52] Goudeau D. M. Parker C. T. Zhou Y. Sela S. Kroupitski Y. Brandl M. T. (2013). The *Salmonella* transcriptome in lettuce and cilantro soft rot reveals a niche overlap with the animal host intestine. *Appl. Environ. Microbiol.* 79 250–262. 10.1128/AEM.02290-12 23104408 PMC3536078

[B53] Grace D. Mahuku G. Hoffmann V. Atherstone C. Upadhyaya H. D. Bandyopadhyay R. (2015). International agricultural research to reduce food risks: case studies on aflatoxins. *Food Sec.* 7 569–582. 10.1007/s12571-015-0469-2

[B54] Gu G. Cevallos-Cevallos J. M. van Bruggen A. H. C. (2013). Ingress of *Salmonella enterica* Typhimurium into tomato leaves through hydathodes. *PLoS One* 8:e53470. 10.1371/journal.pone.0053470 23320087 PMC3540056

[B55] Gutiérrez-Rodríguez E. Gundersen A. Sbodio A. O. Suslow T. V. (2011). Variable agronomic practices, cultivar, strain source and initial contamination dose differentially affect survival of *Escherichia coli* on spinach. *J. Appl. Microbiol.* 112 109–118. 10.1111/j.1365-2672.2011.05184.x 22040351

[B56] Han S. Micallef S. A. (2014). *Salmonella* Newport and Typhimurium colonization of fruit differs from leaves in various tomato cultivars. *J. Food Prot.* 77 1844–1850. 10.4315/0362-028X.JFP-13-562 25364916

[B57] Han S. Micallef S. A. (2016). Environmental metabolomics of the tomato plant surface provides insights on *Salmonella enterica* colonization. *Appl. Environ. Microbiol.* 82 3131–3142. 10.1128/AEM.00435-16 26994076 PMC4959065

[B58] Hazaei H. Purves R. W. Hughes J. Link W. O’Sullivan D. M. Schulman A. H. (2019). Eliminating vicine and convicine, the main anti-nutritional factors restricting faba bean usage. *Trends Food Sci. Technol.* 91 549–556. 10.1016/j.tifs.2019.07.051

[B59] Hsu C. K. Micallef S. A. (2017). Plant-mediated restriction of *Salmonella enterica* on tomato and spinach leaves colonized with *Pseudomonas* plant growth-promoting rhizobacteria. *Int. J. Food Microbiol.* 16 1–6. 10.1016/j.ijfoodmicro.2017.07.012 28778009

[B60] Hu K. Renly S. Edlund S. Davis M. Kaufman J. (2016). A modeling framework to accelerate food-borne outbreak investigations. *Food Control* 59 53–58. 10.1016/j.foodcont.2015.05.017

[B61] Huang S. W. (2013). *Imports Contribute to Year-Round Fresh Fruit Availability.* Washington, DC: USDA Economic Research Service.

[B62] Hunter P. J. Shaw R. K. Berger C. N. Frankel G. Pink D. Hand P. (2015). Older leaves of lettuce (*Lactuca* spp.) support higher levels of *Salmonella enterica* ser. Senftenberg attachment and show greater variation between plant accessions than do younger leaves. *FEMS Microbiol. Lett.* 362:fnv077. 10.1093/femsle/fnv077 25953858 PMC7613271

[B63] Hussain M. A. Dawson C. O. (2013). Economic impact of food safety outbreaks on food businesses. *Foods* 2 585–589. 10.3390/foods2040585 28239140 PMC5302274

[B64] Jacob C. Melotto M. (2020). Human pathogen colonization of lettuce dependent upon plant genotype and defense response activation. *Front. Plant Sci.* 10:1769. 10.3389/fpls.2019.01769 32082340 PMC7002439

[B65] Jacobs J. L. Sundin G. W. (2001). Effect of solar UV-B radiation on a phyllosphere bacterial community. *Appl. Environ. Microbiol.* 67 5488–5496. 10.1128/AEM.67.12.5488-5496.2001 11722897 PMC93334

[B66] Jay-Russell M. T. (2013). What is the risk from wild animals in food-borne pathogen contamination of plants? *CAB Rev.* 8, 1–16. 10.1079/PAVSNNR20138040

[B67] Jeamsripong S. Chase J. A. Jay-Russell M. T. Buchanan R. L. Atwill E. R. (2019). Experimental in-field transfer and survival of *Escherichia coli* from animal feces to romaine lettuce in Salinas Valley, California. *Microorganisms* 7:408. 10.3390/microorganisms7100408 31569566 PMC6843402

[B68] Jechalke S. Schierstaedt J. Becker M. Flemer B. Grosch R. Smalla K. (2019). *Salmonella* establishment in agricultural soil and colonization of crop plants depend on soil type and plant species. *Front. Microbiol.* 10:967. 10.3389/fmicb.2019.00967 31156568 PMC6529577

[B69] Klerks M. M. Franz E. van Gent-Pelzer M. Zijlstra C. van Bruggen A. H. C. (2007). Differential interaction of *Salmonella enterica* serovars with lettuce cultivars and plant-microbe factors influencing the colonization efficiency. *ISME J.* 1 620–631. 10.1038/ismej.2007.82 18043669

[B70] Korir R. C. Everts K. L. Micallef S. A. (2019). Interactions between *Salmonella enterica* Newport, *Fusarium* spp., and melon cultivars. *Foodborne Pathog. Dis.* [Epub ahead of print]. 31755801 10.1089/fpd.2019.2721

[B71] Korte A. Farlow A. (2013). The advantages and limitations of trait analysis with GWAS: a review. *Plant Methods* 9:29. 10.1186/1746-4811-9-29 23876160 PMC3750305

[B72] Kroupitski Y. Golberg D. Belausov E. Pinto R. Swartzberg D. Granot D. (2009). Internalization of *Salmonella enterica* in leaves is induced by light and involves chemotaxis and penetration through open stomata. *Appl. Environ. Microbiol.* 75 6076–6086. 10.1128/AEM.01084-09 19648358 PMC2753090

[B73] Kroupitski Y. Pinto R. Belausov E. Sela S. (2011). Distribution of *Salmonella typhimurium* in romaine lettuce leaves. *Food Microbiol.* 28 990–997. 10.1016/j.fm.2011.01.007 21569943

[B74] Lapidot A. Yaron S. (2009). Transfer of *Salmonella enterica* serovar Typhimurium from contaminated irrigation water to parsley is dependent on curli and cellulose, the biofilm matrix components. *J. Food Protect.* 72 618–623. 10.4315/0362-028X-72.3.618 19343953

[B75] Li H. Thrash A. Tang J. D. He L. Yan J. Warburton M. L. (2019). Leveraging GWAS data to identify metabolic pathways and networks involved in maize lipid biosynthesis. *Plant J.* 98 853–863. 10.1111/tpj.14282 30742331 PMC6850169

[B76] Lima P. M. São José J. F. B. Andrade N. J. Pires A. C. S. Ferreira S. O. (2013). Interaction between natural microbiota and physicochemical characteristics of lettuce surfaces can influence the attachment of *Salmonella* Enteritidis. *Food Control* 30 157–161. 10.1016/j.foodcont.2012.06.039

[B77] Lindow S. E. Brandl M. T. (2003). Microbiology of the phyllosphere. *Appl. Environ. Microbiol.* 69 1875–1883. 10.1128/AEM.69.4.1875-1883.2003 12676659 PMC154815

[B78] Liu C. Guttieri M. J. Waters B. M. Eskridge K. M. Baenziger P. S. (2019). Selection of bread wheat for low grain cadmium concentration at the seedling stage using hydroponics versus molecular markers. *Crop Sci.* 59 945–956. 10.2135/cropsci2018.08.0484

[B79] López-Gálvez F. Gil M. I. Allende A. (2018). Impact of relative humidity, inoculum carrier and size, and native microbiota on *Salmonella* ser. *Typhimurium* survival in baby lettuce. *Food Microbiol.* 70 155–161. 10.1016/j.fm.2017.09.014 29173622

[B80] Lopez-Velasco G. Sbodio A. Tomás-Callejas A. Wei P. Tan K. H. Suslow T. V. (2012a). Assessment of root uptake and systemic vine-transport of *Salmonella enterica* sv. Typhimurium by melon (*Cucumis melo*) during field production. *Int. J. Food Microbiol.* 158 65–72. 10.1016/j.ijfoodmicro.2012.07.005 22824339

[B81] Lopez-Velasco G. Tydingsa H. A. Boyera R. R. Falkinham J. O. Ponder M. A. (2012b). Characterization of interactions between *Escherichia coli* O157:H7 with epiphytic bacteria *in vitro* and on spinach leaf surfaces. *Int. J. Food Microbiol.* 153 351–357. 10.1016/j.ijfoodmicro.2011.11.026 22177225

[B82] Lopez-Velasco G. Welbaum G. E. Falkinham J. O. Ponder M. A. (2011). Phyllosphere bacterial community structure of spinach (*Spinacia oleracea*) as affected by cultivar and environmental conditions at time of harvest. *Diversity* 3 721–738. 10.3390/d3040721

[B83] Macarisin D. Patel J. Bauchan G. Giron J. A. Ravishankar S. (2013). Effect of spinach cultivar and bacterial adherence factors on survival of *Escherichia coli* O157:H7 on spinach leaves. *J. Food Prot.* 76 1829–1837. 10.4315/0362-028X.JFP-12-556 24215684

[B84] Macarisin D. Patel J. Bauchan G. Giron J. A. Sharma V. K. (2012). Role of curli and cellulose expression in adherence of *Escherichia coli* O157:H7 to spinach leaves. *Foodborne Pathog. Dis.* 9 160–167. 10.1089/fpd.2011.102 22315954

[B85] Marcell L. M. Beattie G. A. (2002). Effect of leaf surface waxes on leaf colonization by *Pantoea agglomerans* and *Clavibacter michiganensis*. *Mol. Plant Microbe Interact.* 15 1236–1244. 10.1094/MPMI.2002.15.12.1236 12481996

[B86] Marin S. Ramos A. J. Cano-Sancho G. Sanchis V. (2013). Mycotoxins: occurrence, toxicology, and exposure assessment. *Food Chem. Toxicol.* 60 218–237. 10.1016/j.fct.2013.07.047 23907020

[B87] Marvasi M. George A. S. Giurcanu M. Hochmuth G. J. Noel J. T. Gause E. (2014a). Effects of nitrogen and potassium fertilization on the susceptibility of tomatoes to post-harvest proliferation of *Salmonella enterica*. *Food Microbiol.* 43 20–27. 10.1016/j.fm.2014.03.017 24929878

[B88] Marvasi M. George A. S. Giurcanu M. C. Hochmuth G. J. Noel J. T. Teplitski M. (2015). Effect of the irrigation regime on the susceptibility of pepper and tomato to post-harvest proliferation of *Salmonella enterica*. *Food Microbiol.* 46 139–144. 10.1016/j.fm.2014.07.014 25475277

[B89] Marvasi M. Hochmuth G. J. Giurcanu M. C. George A. S. Noel J. T. Bartz J. (2013). Factors that affect proliferation of *Salmonella* in tomatoes post-harvest: the roles of seasonal effects, irrigation regime, crop and pathogen genotype. *PLoS One* 8:e80871. 10.1371/journal.pone.0080871 24324640 PMC3851777

[B90] Marvasi M. Noel T. George A. S. Farias M. A. Jenkins K. T. Hochmuth G. (2014b). Ethylene signaling affects susceptibility of tomatoes to *Salmonella*. *Microb. Biotechnol.* 7 545–555. 10.1111/1751-7915.12130 24888884 PMC4265073

[B91] Mitra R. Cuesta-Alonso E. Wayadande A. Talley J. Gilliland S. Fletcher J. (2009). Effect of route of introduction and host cultivar on the colonization, internalization, and movement of the human pathogen *Escherichia coli* O157:H7 in spinach. *J. Food Prot.* 72 1521–1530. 10.4315/0362-028x-72.7.1521 19681281

[B92] Moghimi A. Yang C. Anderson J. A. Reynolds S. K. (2019). “Selecting informative spectral bands using machine learning techniques to detect *Fusarium* head blight in wheat,” in *Proceedings of the 2019 ASABE Annual International Meeting*, (St. Joseph, MI: ASABE).

[B93] Moyne A. L. Blessington T. Williams T. R. Koike S. T. Cahn M. D. Marco M. L. (2019). Conditions at the time of inoculation influence survival of attenuated *Escherichia coli* O157:H7 on field-inoculated lettuce. *Food Microbiol.* 85:103274. 10.1016/j.fm.2019.103274 31500714

[B94] Nguyen V. D. Bennett S. D. Mungai E. Gieraltowski L. Hise K. Gould L. H. (2015). Increase in multistate foodborne disease outbreaks–United States, 1973–2010. *Foodborne Pathog. Dis.* 12 867–872. 10.1089/fpd.2014.1908 26284611 PMC11290059

[B95] Noel J. T. Arrach N. Alagely A. McClelland M. Teplitski M. (2010). Specific responses of *Salmonella enterica* to tomato varieties and fruit ripeness identified by *in vivo* expression technology. *PLoS One* 5:e12406. 10.1371/journal.pone.0012406 20824208 PMC2930847

[B96] Oblessuc P. R. Bisneta M. V. Melotto M. (2019). Common and unique Arabidopsis proteins involved in stomatal susceptibility to *Salmonella enterica* and *Pseudomonas syringae*. *FEMS Microbiol. Lett.* 366:fnz197. 10.1093/femsle/fnz197 31529017 PMC7962777

[B97] Oblessuc P. R. Matiolli C. V. Melotto M. (2020). Novel molecular components involved in callose-mediated Arabidopsis defense against *Salmonella enterica* and *Escherichia coli* O157:H7. *BMC Plant Biol.* 20:16. 10.1186/s12870-019-2232-x 31914927 PMC6950905

[B98] Ottesen A. R. González Peña A. White J. R. Pettengill J. B. Li C. Allard S. (2013). Baseline survey of the anatomical microbial ecology of an important food plant: *Solanum lycopersicum* (tomato). *BMC Microbiol.* 13:114. 10.1186/1471-2180-13-114 23705801 PMC3680157

[B99] Painter J. A. Hoekstra R. M. Ayers T. Tauxe R. V. Braden C. R. Angulo F. J. (2013). Attribution of foodborne illnesses, hospitalizations, and deaths to food commodities by using outbreak data, United States, 1998–2008. *Emerg. Infect. Dis.* 19 407–415. 10.3201/eid1903.111866 23622497 PMC3647642

[B100] Pang H. Lambertini E. Buchanan R. L. Schaffner D. W. Pradhan A. K. (2017). Quantitative microbial risk assessment for *Escherichia coli* O157:H7 in fresh-cut lettuce. *J. Food Prot.* 80 302–311. 10.4315/0362-028X.JFP-16-246 28221978

[B101] Pekar J. J. Murray S. C. Isakeit T. S. Scully B. T. Guo B. Knoll J. E. (2019). Evaluation of elite maize inbred lines for reduced *Aspergillus flavus* infection, aflatoxin accumulation, and agronomic traits. *Crop Sci.* 59 2562–2571. 10.2135/cropsci2019.04.0206

[B102] Petersen S. Lyerly J. H. McKendry A. L. Islan M. S. Brown-Guedira G. Cowger C. (2016). Validation of fusarium head blight resistance QTL in US winter wheat. *Crop Sci.* 57 1–12. 10.2135/cropsci2015.07.0415 25388142

[B103] Potnis N. Colee J. Jones J. B. Barak J. D. (2015). Plant pathogen-induced water-soaking promotes *Salmonella enterica* growth on tomato leaves. *Appl. Environ. Microbiol.* 81 8126–8134. 10.1128/AEM.01926-15 26386057 PMC4651078

[B104] Potnis N. Soto-Arias J. P. Cowles K. N. van Bruggen A. H. C. Jones J. B. Barak J. D. (2014). *Xanthomonas perforans* colonization influences *Salmonella enterica* in the tomato phyllosphere. *Appl. Environ. Microbiol.* 80 3173–3180. 10.1128/AEM.00345-14 24632252 PMC4018908

[B105] Poza-Carrion C. Suslow T. Lindow S. (2013). Resident bacteria on leaves enhance survival of immigrant cells of *Salmonella enterica*. *Phytopathology* 103 341–351. 10.1094/PHYTO-09-12-0221-FI 23506362

[B106] Purugganan M. D. (2019). Evolutionary insights into the nature of plant domestication. *Curr. Biol.* 29 R705–R714. 10.1016/j.cub.2019.05.053 31336092

[B107] Quilliam R. S. Williams A. P. Jones D. L. (2012). Lettuce cultivar mediates both phyllosphere and rhizosphere activity of *Escherichia coli* O157:H7. *PLoS One* 7:e33842. 10.1371/journal.pone.0033842 22439006 PMC3306295

[B108] Rastogi G. Sbodio A. Tech J. J. Suslow T. V. Coaker G. L. Leveau J. H. J. (2012). Leaf microbiota in an agroecosystem: spatiotemporal variation in bacterial community composition on field-grown lettuce. *ISME J.* 6 1812–1822. 10.1038/ismej.2012.32 22534606 PMC3446804

[B109] Ribera L. A. Palma M. A. Paggi M. Knutson R. Masabni J. G. Anciso J. (2012). Economic analysis of food safety compliance costs and foodborne illness outbreaks in the United States. *Horttechnology* 22 150–156. 10.21273/HORTTECH.22.2.150

[B110] Roy D. Melotto M. (2019). Stomatal response and human pathogen persistence in leafy greens under preharvest and postharvest environmental conditions. *Postharvest Biol. Technol.* 148 76–82. 10.1016/j.postharvbio.2018.10.013

[B111] Roy D. Panchal S. Rosa B. A. Melotto M. (2013). *Escherichia coli* O157:H7 induces stronger plant immunity than *Salmonella enterica* Typhimurium SL1344. *Phytopathology* 103 326–332. 10.1094/PHYTO-09-12-0230-FI 23301812 PMC3982233

[B112] Sáiz J. Montealegre C. Marina M. García-Ruiz C. (2013). Peanut allergens: an overview. *Crit. Rev. Food Sci. Nutr.* 53 722–737. 10.1080/10408398.2011.556758 23638932

[B113] Sapers G. M. Doyle M. P. (2014). “Chapter 1 - Scope of the produce contamination problem,” in *The Produce Contamination Problem: Causes and Solutions*, 2nd Edn, eds Matthews K. R. Sapers G. M. Gerba C. P. (Amsterdam: Elsevier).

[B114] Schaafsma A. W. (2002). “Economic changes imposed by mycotoxins in food grains: case study of deoxynivalenol in winter wheat,” in *Mycotoxins and Food Safety Advances in Experimental Medicine and Biology*, Vol. 504 eds DeVries J. W. Trucksess M. W. Jackson L. S. (Boston, MA: Springer). 10.1007/978-1-4615-0629-4_28 11922094

[B115] Schikora A. Virlogeux-Payant I. Bueso E. Garcia A. V. Nilau T. Charrier A. (2011). Conservation of *Salmonella* infection mechanisms in plants and animals. *PLoS One* 6:e24112. 10.1371/journal.pone.0024112 21915285 PMC3167816

[B116] Senyuva H. Z. Baricevic Jones I. Sykes M. Baumgartner S. (2019). A critical review of the specifications and performance of antibody and DNA-based methods for detection and quantification of allergens in foods. *Food Addit. Contam. Part A* 36 507–547. 10.1080/19440049.2019.1579927 30856064

[B117] Seo S. Matthews K. R. (2012). Influence of the plant defense response to *Escherichia coli* O157:H7 cell surface structures on survival of that enteric pathogen on plant surfaces. *Appl. Environ. Microbiol.* 78 5882–5889. 10.1128/AEM.01095-12 22706044 PMC3406135

[B118] Siegel K. R. Ali M. K. Srinivasiah A. Nugent R. A. Naraya K. M. V. (2014). Do we produce enough fruits and vegetables to meet global health need? *PLoS One* 9:e104059. 10.1371/journal.pone.0104059 25099121 PMC4123909

[B119] Simko I. Zhou Y. Brandl M. T. (2015). Downy mildew disease promotes the colonization of romaine lettuce by *Escherichia coli* O157:H7 and *Salmonella enterica*. *BMC Microbiol.* 15:19. 10.1186/s12866-015-0360-5 25648408 PMC4334606

[B120] Slayton R. B. Turabelidze G. Bennett S. D. Schwensohn C. A. Yaffee A. Q. Khan F. (2011). Outbreak of Shiga Toxin-producing *Escherichia coli* (STEC) O157:H7 associated with romaine lettuce consumption, 2011. *PLoS One* 8:e55300. 10.1371/journal.pone.0055300 23390525 PMC3563629

[B121] Smale M. Day-Rubenstein K. (2002). The demand for crop genetic resources: international use of the us national plant germplasm system. *World Dev.* 30 1639–1655. 10.1016/S0305-750X(02)00055-4

[B122] Stine S. W. Song I. Choi C. Y. Gerba C. P. (2005). Effect of relative humidity on preharvest survival of bacterial and viral pathogens on the surface of cantaloupe, lettuce, and bell peppers. *J. Food Prot.* 68 1352–1358. 10.4315/0362-028x-68.7.1352 16013370

[B123] Tanksley S. D. McCouch S. R. (1997). Seed banks and molecular maps: unlocking genetic potential from the wild. *Science* 277 1063–1066. 10.1126/science.277.5329.1063 9262467

[B124] Taylor E. V. Nguyen T. A. Machesky K. D. Koch E. Sotir M. J. Bohm S. R. (2013). Multistate outbreak of *Escherichia coli* O145 infections associated with romaine lettuce consumption, 2010. *J. Food Prot.* 76 939–944. 10.4315/0362-028X.JFP-12-503 23726187

[B125] Thilmony R. Underwood W. He S. Y. (2006). Genome-wide transcriptional analysis of the *Arabidopsis thaliana* interaction with the plant pathogen *Pseudomonas syringae* pv. *tomato* DC3000 and the human pathogen *Escherichia coli* O157:H7. *Plant J.* 46 34–53. 10.1111/j.1365-313X.2006.02725.x 16553894

[B126] Thrash A. Tang J. D. DeOrnellis M. Peterson D. G. Warburton M. L. (2020). PAST: the pathway association studies tool to infer biological meaning from GWAS datasets. *Plants* 9:58. 10.3390/plants9010058 31906457 PMC7020396

[B127] Turner K. Moua C. N. Hajmeer M. Barnes A. Needham M. (2019). Overview of leafy greens–related food safety incidents with a California link: 1996 to 2016. *J. Food Prot.* 82 405–414. 10.4315/0362-028X.JFP-18-316 30794462

[B128] Tuttle J. Gomez T. Doyle M. P. Wells J. G. Zhao T. Tauxe R. V. (1999). Lessons from a large outbreak of *Escherichia coli* O157:H7 infections: insights into the infectious dose and method of widespread contamination of hamburger patties. *Epidemiol. Infect.* 122 185–192. 10.1017/s0950268898001976 10355781 PMC2809605

[B129] U.S. Department of Health and Human Services-U.S. Department of Agriculture [HHS-USDA] (2015). *2015–2020 Dietary Guidelines for Americans*, 8th Edn Washington, DC: U.S. Department of Health and Human Services.

[B130] U.S. Food and Drug Administration [FDA] (2018). *Environmental Assessment of Factors Potentially Contributing to the Contamination of Romaine Lettuce Implicated in a Multi-State Outbreak of E. coli O157:H7.* Available online at: https://www.fda.gov/food/outbreaks-foodborne-illness/environmental-assessment-factors-potentially-contributing-contamination-romaine-lettuce-implicated (accessed January 24, 2020).

[B131] U.S. Food and Drug Administration [FDA] (2020). *Statement on the Salinas-Linked Romaine Lettuce E. coli O157:H7 Outbreak and Status Update on Investigation.* Available online at: https://www.fda.gov/news-events/press-announcements/statement-salinas-linked-romaine-lettuce-e-coli-o157h7-outbreak-and-status-update-investigation (accessed January 24, 2020).

[B132] United Nations [UN], Department of Economic, and Social Affairs, Population Division. (2019a). *World Population Prospects 2019: Highlights (ST/ESA/SER.A/423).* Available online at: https://population.un.org/wpp/Publications/Files/WPP2019_Highlights.pdf (accessed January 24, 2020)

[B133] United Nations [UN], Department of Economic, and Social Affairs, Population Division (2019b). *World Urbanization Prospects 2018: Highlights (ST/ESA/SER.A/421).* Available online at: https://population.un.org/wup/Publications/Files/WUP2018-Highlights.pdf (accessed January 24, 2020)

[B134] Uyttendaele M. De Boeck E. Jacxsen L. (2016). Challenges in food safety as part of food security: lessons learnt on food safety in a globalized world. *Procedia Food Sci.* 6 16–22. 10.1016/j.profoo.2016.02.003

[B135] Wang X. Arora R. Horner H. T. Krebs S. L. (2008). Structural adaptations in overwintering leaves of thermonastic and nonthermonastic *Rhododendron* species. *J. Am. Soc. Hortic. Sci.* 133 768–776. 10.21273/JASHS.133.6.768

[B136] Warburton M. L. Williams W. P. (2016). “Advances in mycotoxin-resistant maize varieties,” in *Achieving sustainable maize cultivation: From improved varieties to local applications*, Vol. 1 ed. Watson D. (Sawston: Burleigh Dodds Science Publishing Ltd).

[B137] Wiedemann A. Virlogeux-Payant I. Chaussé A. M. Schikora A. Velge P. (2015). Interactions of *Salmonella* with animals and plants. *Front. Microbiol.* 5:791 10.3389/fmicb.2014.00791 PMC4301013 25653644

[B138] Williams T. R. Moyne A. L. Harris L. J. Marco M. L. (2013). Season, irrigation, leaf age, and *Escherichia coli* inoculation influence the bacterial diversity in the lettuce phyllosphere. *PLoS One* 8:e68642. 10.1371/journal.pone.0068642 23844230 PMC3699665

[B139] Williams W. P. Windham G. L. (2012). Registration of Mp718 and Mp719 germplasm lines of maize. *J. Plant Regist.* 6 200–202. 10.3198/jpr2011.09.0489crg

[B140] Wong C. W. Y. Wang S. Lévesque R. C. Goodridge L. Delaquis P. (2019). Fate of 43 *Salmonella* strains on lettuce and tomato seedlings. *J. Food Prot.* 82 1045–1051. 10.4315/0362-028X.JFP-18-435 31124714

[B141] World Health Organization [WHO] (2015). *WHO Estimates of the Global Burden of Foodborne Diseases: Foodborne Disease Burden Epidemiology Reference Group 2007-2015.* Geneva: World Health Organization.

[B142] Wu F. (2007). Measuring the economic impacts of *Fusarium* toxins in animal feeds. *Anim. Feed Sci. Technol.* 137 363–374. 10.1016/j.anifeedsci.2007.06.010

[B143] Yu Y. C. Yum S. J. Jeon D. Y. Jeong H. G. (2018). Analysis of the microbiota on lettuce (*Lactuca sativa* L.) cultivated in South Korea to identify foodborne pathogens. *J. Microbiol. Biotechnol.* 28 1318–1331. 10.4014/jmb.1803.03007 30301312

[B144] Zain M. E. (2011). Impact of mycotoxins on humans and animals. *J. Saudi Chem. Soc.* 15 129–144. 10.1016/j.jscs.2010.06.006

[B145] Zhou Y. Wang J. Yang X. Lin D. Gao Y. Su Y. (2013). Peanut allergy, allergen composition, and methods of reducing allergenicity: a review. *Int. J. Food Sci.* 2013:909140. 10.1155/2013/909140 26904614 PMC4745518

